# Estimation of HIV Incidence in a Large, Community-Based, Randomized Clinical Trial: NIMH Project Accept (HIV Prevention Trials Network 043)

**DOI:** 10.1371/journal.pone.0068349

**Published:** 2013-07-11

**Authors:** Oliver Laeyendecker, Estelle Piwowar-Manning, Agnes Fiamma, Michal Kulich, Deborah Donnell, Deb Bassuk, Caroline E. Mullis, Craig Chin, Priscilla Swanson, John Hackett, William Clarke, Mark Marzinke, Greg Szekeres, Glenda Gray, Linda Richter, Michel W. Alexandre, Suwat Chariyalertsak, Alfred Chingono, David D. Celentano, Stephen F. Morin, Michael Sweat, Thomas Coates, Susan H. Eshleman

**Affiliations:** 1 Laboratory of Immunoregulation, NIAID, NIH, Baltimore, Maryland, United States of America; 2 Department of Medicine, Johns Hopkins University School of Medicine, Baltimore, Maryland, United States of America; 3 Department of Pathology, Johns Hopkins University School of Medicine, Baltimore, Maryland, United States of America; 4 Program in Global Health, University of California Los Angeles, Los Angeles, California, United States of America; 5 Department of Probability and Statistics, Faculty of Mathematics and Physics, Charles University in Prague, Prague, Czech Republic; 6 Vaccine and Infectious Disease Division, Fred Hutchinson Cancer Research Center, Seattle, Washington, United States of America; 7 Department of Global Health, University of Washington, Seattle, Washington, United States of America; 8 Infectious Diseases Research, Abbott Diagnostics, Abbott Park, Illinois, United States of America; 9 Perinatal HIV Research Unit, Chris Hani Baragwanath Hospital/University of the Witwatersrand, Johannesburg, South Africa; 10 Human Sciences Research Council, Durban, South Africa; 11 Department of Microbiology and Immunology, Muhimbili University of Health and Allied Sciences, Dar es Salaam, Tanzania; 12 Research Institute for Health Sciences, Chiang Mai University, Chiang Mai, Thailand; 13 Department of Psychiatry, School of Medicine, University of Zimbabwe, Harare, Zimbabwe; 14 Department of Epidemiology, Johns Hopkins University Bloomberg School of Public Health, Baltimore, Maryland, United States of America; 15 Department of Medicine, University of California San Francisco, San Francisco, California, United States of America; 16 Department of Psychiatry and Behavioral Sciences, The Medical University of South Carolina, Charleston, South Carolina, United States of America; Centers for Disease Control and Prevention, United States of America

## Abstract

**Background:**

National Institute of Mental Health Project Accept (HIV Prevention Trials Network [HPTN] 043) is a large, Phase III, community-randomized, HIV prevention trial conducted in 48 matched communities in Africa and Thailand. The study intervention included enhanced community-based voluntary counseling and testing. The primary endpoint was HIV incidence, assessed in a single, cross-sectional, post-intervention survey of >50,000 participants.

**Methods:**

HIV rapid tests were performed in-country. HIV status was confirmed at a central laboratory in the United States. HIV incidence was estimated using a multi-assay algorithm (MAA) that included the BED capture immunoassay, an avidity assay, CD4 cell count, and HIV viral load.

**Results:**

Data from Thailand was not used in the endpoint analysis because HIV prevalence was low. Overall, 7,361 HIV infections were identified (4 acute, 3 early, and 7,354 established infections). Samples from established infections were analyzed using the MAA; 467 MAA positive samples were identified; 29 of those samples were excluded because they contained antiretroviral drugs. HIV prevalence was 16.5% (range at study sites: 5.93% to 30.8%). HIV incidence was 1.60% (range at study sites: 0.78% to 3.90%).

**Conclusions:**

In this community-randomized trial, a MAA was used to estimate HIV incidence in a single, cross-sectional post-intervention survey. Results from this analysis were subsequently used to compare HIV incidence in the control and intervention communities.

**Trial Registration:**

ClinicalTrials.gov NCT00203749

## Introduction

Project Accept (HIV Prevention Trials Network [HPTN] 043) is a large, Phase III, community-randomized, HIV prevention trial conducted in 34 communities in Africa (in Soweto and Vulindlela, South Africa; Tanzania; and Zimbabwe) and 14 communities in Thailand (NCT00203749) [1]–[3]. Communities were matched in pairs; communities in the intervention arm received enhanced community-based voluntary counseling and testing services; control communities received standard clinic-based volunteer counseling and testing services [1]. These strategies were designed to change community norms and reduce risk of HIV acquisition among all community members, whether or not they participated directly in the intervention. The intervention was delivered in each community over three years (January 2006–May 2011). The primary endpoint of the study was HIV incidence, assessed in a single, cross-sectional, post-intervention survey of >50,000 individuals in the study communities (September 2009– July 2011).

To our knowledge, Project Accept is the first randomized clinical trial with a primary cross-sectional HIV incidence endpoint, and is one of the largest randomized clinical trials performed to date. There were several reasons why a single, cross-sectional survey was used to evaluate HIV incidence in Project Accept. First, the study intervention included HIV testing; follow-up of an HIV-uninfected cohort for HIV acquisition would have required HIV testing in both control and intervention communities which would have confounded the ability to detect the effect of the intervention. Second, use of a cross-sectional survey allowed HIV incidence to be assessed in a larger, more representative portion of the study population. Third, this approach avoided the bias that may be associated with enrolling HIV-uninfected participants and following them over time (e.g., from the Hawthorne effect [4] or differential participant retention in different study arms).

While there were inherent advantages to using a cross-sectional approach to determine HIV incidence in Project Accept, this also presented significant challenges. When the trial was originally designed, the study plan was to assess HIV incidence using a single HIV incidence assay, the BED capture immunoassay (BED-CEIA) [5]. However, as the trial was being implemented in the field, it became increasingly clear that the BED-CEIA significantly overestimated HIV incidence in some settings. In 2006, the Joint United Nations Programme on HIV/AIDS issued a statement recommending against use of the BED-CEIA for HIV incidence assessments [6]. Since there were no available methods that could provide accurate cross-sectional HIV incidence estimates, the HPTN Network Laboratory and the Project Accept statisticians took on the challenge of developing alternate methods for cross-sectional HIV incidence estimation (see below). The Project Accept trial also presented significant challenges related to the size of the study. New methods were developed to manage the large number of study samples and to confirm the accuracy of HIV testing performed at local in-country laboratories. A pilot study that included approximately 2,500 participants was conducted at additional, non-study communities across the five study sites to develop, test, and optimize these procedures before they were implemented for the primary study endpoint assessment [7].

The methods developed for cross-sectional HIV incidence estimation in Project Accept are based on use of a multi-assay algorithm (MAA) to identify infections that are potentially recent (i.e., MAA positive) [4]. A MAA developed for cross-sectional HIV incidence in subtype B epidemics has been shown to provide accurate incidence estimates in clinical cohort studies performed in the United States [4], [8]–[10]. That MAA uses four biomarkers for HIV incidence estimation: two serologic biomarkers (the BED-CEIA and an avidity assay [11]) and two non-serologic biomarkers (CD4 cell count and HIV viral load). These assays can be performed using in a hierarchical approach to reduce the cost and effort required for the analysis [12].

This report describes the methods used to determine HIV prevalence and HIV incidence in Project Accept. It includes a description of in-country testing of study samples, and quality control testing performed at the HPTN Network Laboratory to confirm the accuracy of in-country test results and determine/confirm the HIV status of study participants. Laboratory data were used to determine HIV prevalence and incidence. Because HIV prevalence was lower than expected at the site in Thailand, HIV incidence was estimated only for the four African sites. HIV incidence was estimated using a MAA that included the same four biomarkers as the MAA developed for subtype B (BED-CEIA, avidity assay, CD4 cell count, and HIV viral load), but used assay cutoffs that were optimized for analysis of HIV incidence in populations with HIV subtypes that are prevalent in South Africa, Zimbabwe, and Tanzania [13]–[16]. The overwhelming majority of HIV infections in South Africa and Zimbabwe are subtype C; in Tanzania, while most infections are subtype C, subtype A and D infections are also observed [16]. The BED-CEIA and the avidity assay used in the MAA frequently misclassify individuals with long-standing subtype D HIV infection as potentially recently infected [17], [18]. To address this, a subset of the Project Accept samples from Tanzania was subtyped to determine the prevalence of subtype D in those communities and assess its potential impact on incidence estimates. This report presents incidence estimates for the Project Accept sites in Africa. The primary result of the Project Accept trial (comparison of HIV incidence in control vs. intervention communities) is presented in a separate report [19].

## Materials and Methods

### Ethics Statement

Written informed consent was obtained from all participants for participation in Project Accept. Human experimentation guidelines of the US Department of Health and Human Services and those of the authors’ institution(s) were followed in the conduct of this research. The Project Accept study was approved by ethical review committees for the study sites (South Africa: the University of the Witwatersrand, Human Research Ethics Committee [Medical]; Tanzania: the Muhimbili University of Health and Allied Sciences [MUHAS] Senate Research and Publication Committee and the National Institute of Medical Research Ethics Committee; Zimbabwe: the Medical Research Council of Zimbabwe; Thailand: the Ministry of Public Health Ethical Review Committee of Research in Human Subjects [MOPHEC] and the Human Experimentation Committee Research Institute for Health Sciences, Chiang Mai University), and at collaborating institutions in the United States (the Committee on Human Research, University of California, San Francisco; the UCLA Office of the Human Research Protection Program (OHRPP); the Johns Hopkins University Bloomberg School of Public Health Institutional Review Board; the Medical University of South Carolina, Institutional Review Board for Human Research).

### Source of Study Samples

Criteria for inclusion of participants in the post-intervention assessment survey of Project Accept are described elsewhere [1]. Briefly, participants were selected from all community residents by randomly sampling households in the study community. All eligible household members aged 18–32 years were invited to participate. Each participant provided blood samples for in-country HIV testing and CD4 cell count testing. Stored plasma aliquots were used for further analyses.

### In-country Testing

HIV testing was performed at local laboratories, as previously described [7]. Two HIV rapid tests were performed in parallel. Samples were classified as HIV POS (both rapid tests reactive), HIV DISC (one rapid test reactive, one rapid test non-reactive), or HIV NEG (both rapid tests non-reactive). These terms (HIV POS, HIV DISC, HIV NEG) were used to distinguish between the initial classification of samples based solely on HIV rapid testing and the final classification of HIV infection status. A CD4 cell count was performed for all HIV POS and HIV DISC samples.

### HPTN Network Laboratory Testing

Samples were shipped to the HPTN Network Laboratory at the Johns Hopkins University School of Medicine (Baltimore, MD) for further testing. Quality assurance testing was performed to confirm the results of in-country HIV testing and to determine the HIV status of HIV DISC samples; assays used for this testing included: the Vitros EIA Human Immunodeficiency Virus Type 1 and/or 2 (HIV-1/2) Antibody Detection in Human Serum and Plasma (VITROS ECi/ECiQ Immunodiagnostic System, Ortho Diagnostics, Johnson & Johnson, Pencoed, United Kingdom), the Genetics System HIV-1 Western Blot (BioRad Laboratories, Redmond WA), and the APTIMA® HIV-1 RNA Qualitative Assay (Gen-Probe Inc., San Diego, CA). A subset of the HIV NEG samples was tested using the ARCHITECT® HIV Ag/Ab Combo assay (HIV Combo; List: 2P36; Abbott Diagnostics, Wiesbaden, Germany). The HIV Combo assay was performed only once; reactive samples were evaluated further using additional assays listed above.

Further testing to assess HIV incidence was performed using the BED-CEIA (Calypte Biomedical Corporation, Lake Oswego, OR) [5] and an avidity assay based on the Genetic Systems HIV-1/HIV-2 Plus O EIA (Bio-Rad Laboratories, Redmond, WA) [11]. Results from the BED-CEIA and avidity assays were reported as normalized optical density units (OD-n) and avidity index (%), respectively. Viral load testing was performed for a subset of study samples (Roche AMPLICOR HIV-1 MONITOR test, version 1.5, Roche Diagnostics, Indianapolis, IN). Samples were considered to be MAA positive if they had all of the following test results: BED-CEIA <1.2 OD-n, avidity index <90%, CD4 cell count >200 cells/mm^3^, and HIV viral load >400 copies/ml; this MAA has a mean window period of 259 days in subtype A and C epidemics [14]. HIV subtypes were determined for a subset of samples from Tanzania by phylogenetic analysis of HIV *gag* and gp41 sequences (GenBank accession numbers: KC589446–KC589569 [*Gag*] and KC589570–KC589696 [gp41]) [20].

Selected plasma samples were tested for the presence of ARV drugs [21]. Samples were analyzed using a qualitative, high-resolution accurate mass spectrometric method developed to detect 15 ARV drugs, including protease inhibitors (PIs: amprenavir, atazanavir, darunavir, indinavir, lopinavir, nelfinavir, ritonavir, saquinavir, and tipranavir), non-nucleotide reverse transcriptase inhibitors (NNRTIs: efavirenz, nevirapine), and nucleoside/nucleotide reverse transcriptase inhibitors (NRTIs: emtricitabine, lamivudine, tenofovir). Detailed methods are described in the legend for Tables S1 and S2 in File S1.

### Data Management

The Laboratory Data Management System (LDMS, Frontier Science & Technology Research Foundation, Inc.) was used to track specimen collection, testing, storage, and shipping. Data from study sites and in-country laboratories were submitted to the Project Accept Statistical Center. After those data were merged and reviewed, they were submitted to the HPTN Statistical and Data Management Center (SDMC). Data from the HPTN Network Laboratory were submitted directly to the HPTN SDMC.

## Results

### In-country Testing

In Project Accept, 46,693 (94.4%) of 49,461 eligible participants provided blood samples for analysis (Table 1). Samples were initially classified as HIV POS, HIV DISC, or HIV NEG based on results from two HIV rapid tests (see Methods). CD4 cell count testing was performed for HIV POS and HIV DISC samples. Overall, 7,724 samples were HIV POS, 274 were HIV DISC, and 38,695 were HIV NEG (Table 1). The initial prevalence estimate based on in-country testing was 16.5% (1.02% in Thailand, 5.99% to 30.8% at the four African sites, Table 1). All HIV POS and HIV DISC samples and a subset of HIV NEG samples were shipped to the HPTN Network Laboratory for further analysis. The number of incident infections identified in Thailand was too low to contribute to the endpoint assessment (3 incident infections in 14 matched communities). Therefore, the endpoint assessment was limited to the four African sites.

**Table 1:** Sample collection and in-country laboratory analysis.

	Thailand	Tanzania	Zimbabwe	Soweto South Africa	Vulindlela South Africa	Africa (4 sites)
Eligible participants^a^	8,041	9,974	12,666	14,682	12,139	49,461
Participants with blood samples^b^	7,619	9,041	11,880	13,929	11,843	46,693
HIV NEG	7,502	8,312	10,313	11,922	8,148	38,695
HIV DISC	39	187	19	22	46	274
HIV POS	78	542	1,548	1,985	3,649	7,724
Initial estimate of HIV prevalence^c^	1.02%	5.99%	13.0%	14.3%	30.8%	16.5%

a Excludes participants who were not contacted, declined participation, or did not meet enrollment criteria.

b Samples were not obtained for 2,744 eligible participants (2,310 no consent, 439 blood draw failure, 19 excluded for other reasons). The HIV status of study participants was initially characterized based on the results of the two HIV rapid tests performed in-country (see Methods): HIV POS: two reactive HIV rapid tests. HIV DISC: one reactive and one non-reactive HIV rapid test. HIV NEG: two non-reactive HIV rapid tests.

c An initial estimate of HIV prevalence was based on in-country testing (calculated as # HIV POS samples/total # samples×100).

### Analysis of HIV NEG Samples

**Figure 1:**
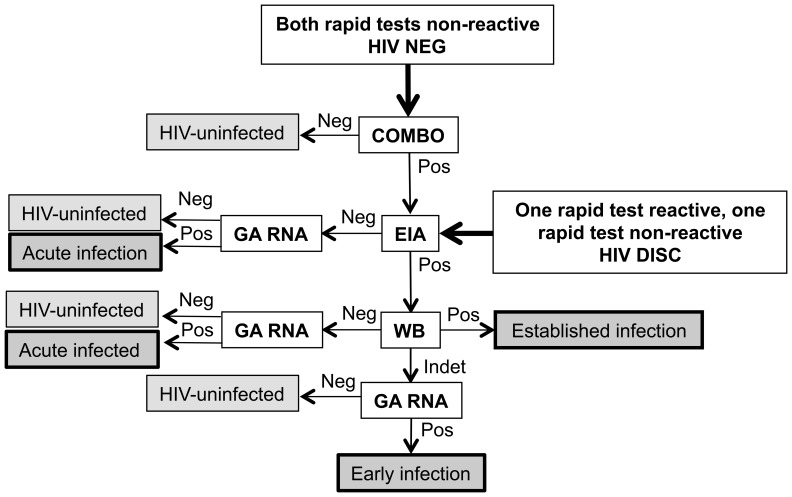
Algorithms used for quality assurance testing of study samples. The figure illustrates the testing algorithms that were used to determine and/or confirm the HIV status of study samples. This quality assurance testing was performed at the HPTN Network Laboratory (see Methods). The algorithm used for quality assurance testing was determined by results obtained from HIV rapid testing performed at the study sites (for samples initially designated as HIV NEG, HIV DISC, and HIV POS, see Methods). Quality assurance testing was performed for HIV POS samples if results from the avidity assay suggested absent or very low levels of anti-HIV antibodies (weird avidity). In this case, the HIV DISC algorithm was used to determine HIV status. Neg indicates that a negative or non-reactive test result was obtained. Pos indicates that a positive or reactive test result was obtained. Arrows (non-bolded) indicate the next step in sample testing. The following abbreviations were used to describe assays and tests used in the analysis (see Methods): HIV Combo: ARCHITECT® HIV Ag/Ab Combo assay; EIA: Vitros EIA Human Immunodeficiency Virus Type 1 and/or 2 (HIV-1/2) Antibody Detection in Human Serum and Plasma; GA RNA: APTIMA® HIV-1 RNA Qualitative Assay; WB: Genetics System HIV-1 Western Blot.

**Figure 2:**
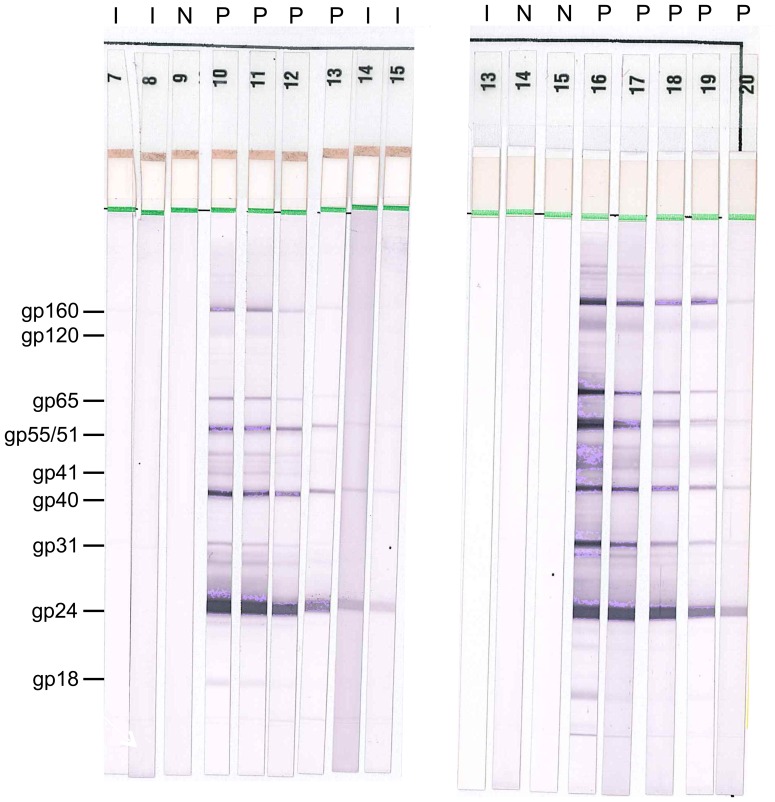
Investigation of sample cross-contamination at a study site. The figure shows two examples of results from two Western blot runs that were performed at the central laboratory as part of an investigation of discordant test results. Results from various laboratory tests are shown above the Western blot strips. HIV rapid tests were performed at a laboratory at the study site in Soweto, South Africa using whole blood; N indicates that both rapid tests were non-reactive, R indicates that both rapid tests were reactive. Samples were subsequently processed to produce plasma aliquots for storage which were later shipped to a central laboratory in the United States for analysis. Results from the ARCHITECT Combo HIV Ag/Ab test are shown (COMBO); N indicates that the Combo test was non-reactive, R indicates the Combo test was reactive. Samples were also tested using the Vitros EIA Human Immunodeficiency Virus Type 1 and/or 2 (HIV-1/2) Antibody Detection in Human Serum and Plasma (EIA); N indicates that the EIA test was non-reactive, R indicates the EIA test was reactive. Western blots were interpreted as negative (N) or positive (P) based on the pattern of bands observed. The banding pattern typically varies among different HIV-positive samples. The panel on the left shows that samples 11–15 were likely to have been cross-contaminated by transfer of plasma from sample 10 into those samples during aliquot preparation (sequential unintended transfer of plasma from tube to tube). Similar findings are shown in the panel on the right; samples 17–19 were likely to have been cross-contaminated by transfer of plasma from sample 16 into those samples. Further investigation at the study site confirmed that a technologist working at the study site prepared sample aliquots without changing pipette tips. All of the samples that may have been processed on the days that this technologist was working in the laboratory were excluded from the endpoint analysis.

For quality assurance, the first two batches of HIV NEG samples received at the HPTN Network Laboratory from each study site were tested using the automated, fourth generation HIV screening assay (HIV Combo, see Methods; Figure 1). Non-reactive test results were obtained for 9,631 (98.9%) of the 9,741 HIV NEG samples tested, confirming that the participants were HIV-uninfected; the remaining 110 samples had reactive test results (Table 2). A high proportion of the reactive samples (59% of the 110 samples) were from Soweto (2.24% of the HIV NEG samples from Soweto were reactive, compared to 0.35–0.79% at the other three African study sites). The high frequency of reactive test results from this site prompted an investigation that revealed that a technologist working at site did not change pipette tips while preparing plasma aliquots (Figure 2). Based on these findings, HIV Combo testing was performed for all HIV NEG samples from Soweto that were processed during the period when the technologist was employed in the laboratory. The following approach was used to exclude samples that may have been contaminated: (1) the technologist was on duty that day and at least one HIV NEG sample had a reactive HIV Combo test, or (2) the technologist was not on duty that day (or it could not be determined whether the technologist was on duty), and at least two HIV NEG samples had reactive HIV Combo tests. Note that all samples meeting these criteria were excluded, regardless of their original designation (HIV POS, HIV DISC, or HIV NEG). Overall, this process resulted in excluding 1,710 HIV NEG samples and 292 samples that were either HIV POS or HIV DISC. After excluding for contamination, 58 (0.62%) of 9,314 HIV NEG samples were HIV Combo reactive, including 0.43% of the HIV NEG samples from Soweto. Those samples were analyzed further using the testing algorithm shown in Figure 1; 44 were classified as HIV negative, seven were classified as acute infections (HIV RNA positive, HIV antibody negative), one was classified as early HIV infection (HIV RNA positive, EIA positive, Western blot indeterminate), and six were classified as established HIV infections (Western blot positive). Note that samples originally classified as HIV NEG were analyzed for quality assurance only; results from these samples were not used in the endpoint assessment.

**Table 2:** Quality assurance testing.

	Tanzania	Zimbabwe		Soweto South Africa	Vulindlela South Africa		Total		
**Quality assurance testing of HIV NEG samples** a									
Tested with HIV Combo (%)^b^	1,932		1,999	2,900		2,910			9,741
Combo reactive	15 (0.78%)		7 (0.35%)	65 (2.24%)		23 (0.79%)			110 (1.13%)
**HIV POS and HIV DISC samples censored** (among those originally classified as HIV POS or HIV DISC)									
Excluded due to contamination	0		0	292		0			292
Excluded for other reasons^c^	0		1	23		4			28
**Classification of HIV DISC samples** a									
Samples remaining after exclusions	187		19	20		46			272
HIV uninfected	184		18	18		45			265
HIV infected	3		1	2		1			7
Acute infection	1		0	0		0			1
Early infection	0		0	0		0			0
Established infection	2^d^		1^d^	2^e^		1^e^			6
**Classification of HIV POS samples** a									
Samples remaining after exclusions	542	1,547		1,672		3,645		7,406	
HIV uninfected	9	17		22		4		52	
HIV infected	533	1,530		1,650		3,641		7,354	
Acute infection	0	0		0		3		3	
Early infection	1	0		0		2		3	
Established infection	532	1,530		1,650		3,636		7,348	

Abbreviations: WB: Western blot; HIV Combo: ARCHITECT HIV Ag/Ab Combo assay.

a The HIV status of study participants was initially characterized based on the results of the two HIV rapid tests performed in-country (see Methods): HIV POS: two reactive HIV rapid tests. HIV DISC: one reactive and one non-reactive HIV rapid test. HIV NEG: two non-reactive HIV rapid tests. The testing algorithm used to confirm the HIV status of HIV NEG and HIV DISC samples (quality assurance testing) are shown in Figure 1. Quality assurance testing was only performed for HIV POS samples if results from the avidity assay suggested absent or extremely low levels of anti-HIV antibodies.

b This indicates the number of samples that had reactive results using the HIV Combo assay (signal/cutoff >1). According to the package insert, specimens that are initially reactive with HIV Combo should be retested in duplicate and only repeatedly reactive specimens are considered reactive. In this study, samples were analyzed only once using the HIV Combo assay.

c 28 samples were excluded for reasons other than contamination, including: no CD4 cell count obtained (N = 5); insufficient quantity of plasma stored for testing (N = 2); failure of sample tracking (N = 17); protocol violation (N = 4).

d These three samples were subsequently classified as MAA positive.

e These three samples were subsequently classified as MAA negative.

### Analysis of HIV DISC Samples

A total of 272 HIV DISC samples remained after sample exclusion (see above, Table 2). These samples were analyzed using the testing algorithm shown in Figure 1; 265 (97.4%) of the samples were confirmed to be from individuals who did not have HIV infection. The remaining seven samples included one acute infection and six established infections (Table 2).

### Analysis of HIV POS Samples

Quality assurance testing of HIV POS samples was restricted to samples with avidity assay results that suggested that HIV antibodies were absent or present at very low levels. Those samples were further analyzed using the testing algorithm for HIV DISC samples (Figure 1); 52 of those samples (0.70% of 7,406 HIV POS samples) were from HIV-uninfected individuals. This was consistent with the rate of false positive in-country test results observed in the Project Accept pilot study (0.63%) [7]. The remaining samples included three acute infections, three early infections, and 7,348 established HIV infections (Table 2).

### Analysis of HIV Subtype

We determined the frequency of subtype D infection at the Project Accept site in Tanzania by testing 113 samples from that site (at least 10 samples from each community, see Methods). Overall, 9.7% of the samples were subtype D (with 44.3% subtype A, 22.1% subtype C, and 23.9% intersubtype recombinant). The highest prevalence of subtype D in a single community was 30% (3/10), while three communities had no subtype D HIV among the samples tested. Overall, 534 (7.26%) of the 7,354 established infections in Project Accept were from Tanzania (Table 2). Based on a subtype D prevalence of 9.7%, we estimated that only 0.7% of the established infections in Project Accept would be subtype D. Even if some of the non-recent subtype D infections were misclassified as MAA positive, it would be unlikely to influence overall HIV incidence estimated in the trial. There was no significant difference in the performance of the BED-CEIA and avidity assay with subtypes A and C in our validation studies [15].

### HIV Incidence Assessment

**Figure 3:**
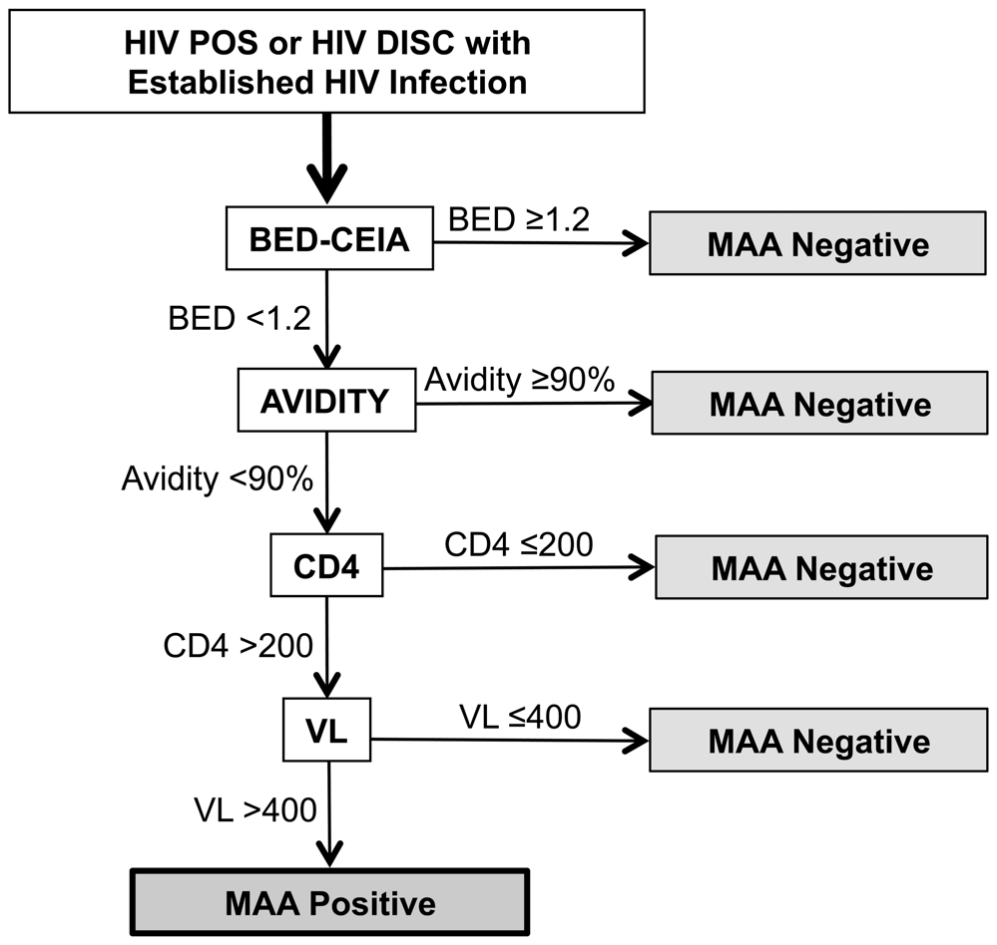
Multi-assay algorithm (MAA) used for HIV incidence estimation. Study samples were initially designated as HIV NEG, HIV DISC, and HIV POS based on HIV rapid testing performed at study sites (see Methods). HIV POS and HIV DISC samples (those that had at least one reactive HIV rapid test) were further evaluated at the HPTN Network Laboratory to determine the HIV status of each sample. The majority of the HIV POS samples and some of the HIV DISC samples were determined to be from individuals with established HIV infection (Table 3). Those samples were analyzed further using a multi-assay algorithm (MAA) developed for HIV incidence estimation. The figure shows the MAA testing schema. Samples were initially tested with the BED capture immunoassay (BED-CEIA) and an avidity assay. Samples that had a BED-CEIA result ≥1.2 normalized optical density units (OD-n) were considered to be MAA negative and were not evaluated further. The remaining samples were evaluated based on results of the avidity assay. Samples that had an avidity assay result (avidity index) ≥90% were considered to be MAA negative and were not evaluated further. The remaining samples were evaluated based on results of CD4 cell count testing that was performed at study sites around the time of sample collection (CD4). Samples that had CD4 cell count result <200 cells/mm^3^ were considered to be MAA negative and were not evaluated further; if a CD4 cell count result was not obtained at the time of sample collection, recency could not be assessed. The remaining samples were tested using an HIV viral load assay (VL). Samples that had a viral load result <400 copies/mL were considered to be MAA negative and were not evaluated further. Samples that met all of the criteria for the MAA (BED-CEIA <1.2 OD-n+avidity index <90%+CD4 cell count >200 cells/mm^3^+ HIV viral load >400 copies/mL) were classified as MAA positive.

Results from in-country and quality assurance testing identified 7,361 confirmed HIV infections in Project Accept, including four acute infections, three early infections, and 7,354 established infections (Table 3). The 7,354 samples from participants with established infections were analyzed using the MAA (see Methods, Figure 3). For this analysis, all 7,354 samples were tested with the BED-CEIA and the avidity assay. HIV viral load testing was only performed for samples that had a BED-CEIA result <1.2 OD-n, an avidity index <90%, and a CD4 cell count >200 cells/mm^3^. This evaluation identified 467 MAA positive samples (Table 3).

**Table 3:** Final sample classification, HIV prevalence, and estimated annual HIV incidence.

	Tanzania	Zimbabwe	Soweto SouthAfrica		VulindlelaSouth Africa		Total/Overall	
**Final sample classification** (among those originally designated as HIV DISC or HIV POS)^a^								
HIV-uninfected	193	35		40		49		317
HIV-infected	536	1,531		1,652		3,642		7,361
Acute infection	**1**	0		0		**3**		**4**
Early infection	**1**	0		0		**2**		**3**
Established infection	534	1,531		1,652		3,637		7,354
MAA negative	479	1,461		1,547		3,400		6,887
MAA positive^b^	55	70		105		237		467
ARV drug(s) detected	10	3		4		12		29
No ARV drugs detected	**45**	**66**		**101**		**220**		**432**
Not tested/no result^c^	**0**	**1**		**0**		**5**		**6**
Total incident infections^d^	47	67		101		230		445
**HIV Prevalence**								
HIV prevalence	5.9%	12.9%		14.1%		30.8%		16.5%
**Annual Incidence Estimate**								
HIV incidence	0.78%	0.91%		1.18%		3.90%		1.60%

Abbreviations: MAA: multi-assay algorithm; ARV: antiretroviral drug.

a The HIV status of study participants was initially characterized based on the results of the two HIV rapid tests performed in-country (see Methods). The testing algorithms used to classify samples according to HIV infection status are shown in Figures 1 and 2.

b Samples classified as MAA positive (see Figure 1) were tested for the presence of ARV drugs (see text).

c Two samples did not have sufficient volume remaining for testing and four samples failed testing; these were included in the analysis as incident infections.

d Incident infections include acute infections, early infections (confirmed infections with indeterminate Western blots), and established infections classified MAA positive (see Figure 2) that either had no ARV drugs detected or no ARV test result (shown in bold).

In resource-limited settings, individuals with recent HIV infection are often not aware of their HIV status; even if they are, they are not likely to initiate ARV treatment until later in the course of their disease. For this reason, ARV treatment has been used as a surrogate marker of long-standing HIV infection in some settings [22]. As a final step in our incidence assessment, samples classified as MAA positive were tested for the presence of ARV drugs; this testing was performed using a high-throughput, qualitative ARV drug screen that includes ARV drugs available in the study countries at the time that the trial was performed (Table 4) [21]. ARV test results were obtained for 461 (98.7%) of the 467 MAA positive samples; 29 (6.7%) of those samples had at least one ARV drug detected and were not included in the primary endpoint assessment, leaving a total of 439 infections classified as incident (Table 3). For comparison, we also tested all 88 samples that were classified as MAA negative solely on the basis of viral suppression (i.e., samples with BED-CEIA <1.2 OD-n, avidity index <90%, CD4 cell count >200 cells/mm^3^, and HIV viral load <400 copies/ml); 57 (64.8%) of the 88 samples in this group had at least one ARV drug detected (Table 4). Detailed results of ARV drug testing are provided in Tables S1 and S2 in File S1.

**Table 4:** Detection of antiretroviral drugs in study samples.

				ARV drugs detected		
Sample type	N	Female		No drugs	1 drug	≥2 drugs
MAA positive (within the windowperiod for recent infection usingthe MAA)	461	311 (67.5%)		432 (93.7%)	26 (5.6%)	3 (1.1%)
MAA negative, excluded from thewindow period for recent infectionbased solely on low viral load	88	65 (73.9%)		31 (64.8%)	12 (13.6)	45 (51.1%)

Abbreviations: ARV: antiretroviral; MAA: multi-assay algorithm; N: number of samples/participants.

Based on the analysis described above, the overall HIV prevalence in Project Accept was 16.5%, with a range from 5.93% in Tanzania to 30.8% in Vulindlela, South Africa (Table 3). These prevalence rates are very similar to the initial prevalence rates determined solely by in-county HIV rapid testing (Table 1). In the HIV incidence assessment, the following infections were classified as incident: acute infections (N = 4), early infections (N = 3) and infections that were classified as MAA positive that either had no ARV drugs detected or had no ARV test result (N = 438); overall, 455 incident infections were identified.

The overall annual HIV incidence in the Project Accept was 1.60%, with a range from 0.78% in Tanzania to 3.90% in Vulindlela, South Africa (Table 3).

## Discussion

The assessment of HIV incidence in Project Accept began by determining the HIV status of each study participant. As a first step, samples were tested in-country using two HIV rapid tests performed in parallel. Quality assurance testing was performed at a central laboratory. In general, quality assurance testing confirmed the results of in-country HIV rapid testing. Notably, quality assurance testing identified a problem with sample processing at one study site that resulted in cross-contamination of numerous sample aliquots. As a result, a significant portion of the samples from the site in Soweto, South Africa was excluded from the endpoint analysis. This finding emphasizes the importance of rigorous quality assurance testing.

Samples that were confirmed to be from participants with HIV infection were further characterized based on the stage of HIV infection (acute, early, or established infection). Acute and early HIV infections were considered to be incident infections. Very few acute HIV infections were identified in this study (among 9,269 samples with non-reactive HIV rapid tests, only 7 acute infections were identified using a 4^th^ generation screening assay). This emphasizes the limitation of using acute HIV infection for incidence determination [23].

Samples from individuals with established infection were analyzed further using a MAA that was optimized for incidence estimation in Project Accept; development and validation of this MAA is described in two previous reports [14], [15]. This MAA has a mean window period of 259 days and was shown in validation studies to provide sufficient power to detect a 35% decrease in HIV incidence in the intervention communities of Project Accept [14], [15]. Our previous reports also demonstrate that this MAA has an overall precision that is comparable or better than incidence estimates based on a simulated cohort follow-up study of 6-months duration [14], [15]. One consideration in the development of this MAA was HIV subtype. Previous studies have shown that individuals with long-standing HIV subtype D infection are frequently misclassified by serologic assays as potentially recently infected [15], [17]. The MAA used in this study was optimized for analysis of HIV incidence in subtypes A and C [14], [15]. Because subtype D HIV is known to circulate in Tanzania, we determined the prevalence of subtype D HIV at the Project Accept site in Tanzania. Fortunately, the prevalence of subtype D at that site was low (9.7%) and was unlikely to impact the incidence assessment in the trial.

Another potential confounder in cross-sectional HIV incidence estimation is antiretroviral treatment (ART). Prolonged ART is associated with down-regulation of the anti-HIV antibody response, which can affect the performance of serologic incidence assays [18], [24]. The MAA used in this study characterizes individuals who are virally suppressed as MAA negative. However, even in the absence of viral suppression, ART may serve as a useful surrogate for non-recent HIV infection, since individuals are unlikely to initiate ART in the first few months after infection. For this reason, we tested samples that were classified as MAA positive for the presence of antiretroviral drugs; 29 (6.7%) of the 467 MAA positive samples had one or more antiretroviral drugs detected and were excluded from the incidence analysis. The infrequent detection of antiretroviral drugs in the MAA positive samples provides further support for use of the MAA to identify recent HIV infection; notably, 64.8% of the samples that met three of the four criteria of the MAA but had undetectable HIV RNA (<400 copies/mL) contained antiretroviral drugs. It is notable that a significant proportion (35.2%) of those did not contain antiretroviral drugs; those samples, which represented 0.42% of the 7,354 samples analyzed with the MAA, were most likely from elite controllers. Note that the MAA classifies all samples with low viral load as MAA negative (regardless of whether antiretroviral drugs are present), which is important, since elite controllers can be misclassified as recently infected using serologic incidence assays [24].

This study provides a model for cross-sectional estimation of HIV incidence in a large, randomized clinical study. This approach is likely to become more widely used, as HIV prevention trials move from longitudinal studies of HIV-uninfected cohorts to larger, community-randomized trials of combination prevention interventions delivered to entire populations. Strict attention to the quality of laboratory test results, and rigorous validation of testing algorithms for cross-sectional incidence estimation using large validation sample sets from relevant study populations are critical elements for this type of analysis.

## Supporting Information

File S1 Table S1, Antiretroviral drugs detected by drug class among samples that were classified as MAA positive using the multi-assay algorithm. Table S2, Antiretroviral drugs detected by drug class among samples that were classified as MAA negative using the multi-assay algorithm solely on the basis of HIV viral load ≤400 copies/ml.
(PDF)
