# Supplementary material for: Estimation of HIV Incidence in a Large, Community-Based, Randomized Clinical Trial: NIMH Project Accept (HIV Prevention Trials Network 043)
Source: PLoS One. 2013 Jul 11;8(7):e68349. doi: 10.1371/journal.pone.0068349 (PMC3708944; doi:10.1371/journal.pone.0068349)
Supplement: File S1 — Table S1, Antiretroviral drugs detected by drug class among samples that were classified as MAA positive using the multi-assay algorithm. Table S2, Antiretroviral drugs detected by drug class among samples that were classified as MAA negative using the multi-assay algorithm solely on the basis of HIV viral load ≤400 copies/ml. (PDF) [file pone.0068349.s001.pdf]

SUPPLEMENTAL TABLES

Legend for Supplementary tables:

Plasma samples were tested for the presence of ARV drugs using a qualitative, high-resolution accurate mass spectrometric (HRAMS) method developed to detect a panel of antiretroviral (ARV) drugs, including protease inhibitors (PIs: amprenavir, atazanavir, darunavir, indinavir, lopinavir, nelfinavir, ritonavir, saquinavir, and tipranavir), non-nucleoside reverse transcriptase inhibitors (NNRTIs: efavirenz, nevirapine), and nucleoside/nucleotide reverse transcriptase inhibitors (NRTIs: emtricitabine, lamivudine, tenofovir). The analytical method was developed to detect the following ARV drugs: 100 µl of ARV drug standards in drug free human serum (BioRad Laboratories, Irvine, CA) or 100 µl subject specimen was combined with 300 µl of acetonitrile-containing internal standard (125 ng/ml morphine-D3), vortexed and centrifuged. Supernatants were evaporated to dryness and reconstituted in water. For analytical separation, 30 µl was injected onto a liquid chromatography system equipped with Transcend pumps (Thermo Fisher Scientific, San Jose, CA). The chromatographic run began with 30 seconds of 100% water containing 0.1% glacial acetic acid (mobile phase A), followed by a 60 second ramp to 10% acetonitrile containing 0.1% glacial acetic acid (mobile phase B). This slow ramp facilitated the elution of water-soluble analytes. The chromatographic separation continued with a step to 15% mobile phase B followed by a ramp to 95% mobile phase B over 600 seconds. Following the elution of all analytes, the column was washed for 60 seconds with a 2:2:1 ratio of isopropanol:acetonitrile:acetone and the column was re-equilibrated for 180 seconds with mobile phase A. The entire chromatographic run occurred at a flow rate of 500 µL/min. Analytes were eluted from a Hypersil Gold PFP 100 x 2.1 mm; 5 µm particle size high performance liquid chromatography (HPLC) column (Thermo Fisher Scientific) and detected over 14.9 minutes using the Exactive Orbitrap mass analyzer (Thermo Fisher Scientific). The mass spectrometer method included two positive-mode scan events: one full scan event with ultra-high resolution

(100,000 @ 1Hz) and one in-source collision-induced dissociation (SCID) event at 45eV with enhanced resolution (25,000 @ 4Hz). Both scan events were programmed for 100 millisecond (msec) maximum injection time and balanced automatic gain control intensity (ACG) targets. The analytical method was found to have a limit of identification of ≤ 10 ng/ml for all ARV drugs, with the exception of efavirenz, which had a limit of identification of 150 ng/ml. Positive identification was determined by exact mass analysis at 5 ppm discrimination, analyte retention time, and the identification of mass transitions when applicable. Carryover studies were performed by adding 2,500 ng/ml of a mixture of all ARV drugs in a specimen, and then running a post-high blank to assess signal intensity. Significant carryover was not detected for any analyte. Data review was performed using a combination of automated review using the ToxID program, which set thresholds based on the presence of fragment, signal intensity and retention time windows, as well as a manual review of purported positives.

Among samples with a single drug detected, 25/38 (65.8%) had an NNRTI, 9/38 (23.7%) had an NRTI, and 4/38 (10.5%) had a PI. Among samples that had two or more ARV drugs detected, all had an NNRTI (either nevirapine or efavirenz) and all but one had the NRTI, lamivudine; a small number of samples had a PI or another NRTI detected. A significantly higher proportion of individuals who were classified as MAA negative solely on the basis of low viral load had at least one ARV drug detected, compared to the MAA positive group (57/88=64.8% vs. 29/461=6.3%, P<0.001). Individuals in the virally suppressed MAA negative group were also more likely to have two classes of ARV drugs detected (45/88=51.1% vs. 3/461=0.65%, P<0.001).

Table S1: Antiretroviral drugs detected by drug class among samples that were classified as MAA positive using the multi-assay algorithm*.

	Any ARV	Any NNRTI	Any NRTI	Any PI	NVP only	EFV only	NNRTI only	NRTI only	PI only	NNRTI + NRTI only	All three classes	No. samples
Total Number	29	22	7	3	4	15	19	4	3	3	0	461
Gender												
Female	17	14	5	1	0	11	11	2	1	3	0	311
Male	12	8	2	2	4	4	8	2	2	0	0	150
Study site												
Zimbabwe	3	1	2	0	1	0	1	2	0	0	0	69
Tanzania	10	10	0	0	0	10	10	0	0	0	0	55
Vulindlela	12	9	2	2	3	5	8	1	2	1	0	232
Soweto	4	2	3	1	0	0	0	1	1	2	0	105
Age												
≤ 25 years	14	10	3	1	4	6	10	3	1	0	0	293
> 25 years	15	12	4	2	0	9	9	1	2	3	0	167
Unknown	0	0	0	0	0	0	0	0	0	0	0	1
Subgroup												
Female ≤ 25	7	6	1	0	0	6	6	1	0	0	0	215
Female > 25	10	8	4	1	0	5	5	1	1	3	0	96
Male Age ≤ 25	7	4	2	1	4	0	4	2	1	0	0	78
Male Age > 25	5	4	0	1	0	4	4	0	1	0	0	71
Male Unknown Age	0	0	0	0	0	0	0	0	0	0	0	1
Past pregnancy												
Yes	0	0	0	0	0	0	0	0	0	0	0	22
No	17	14	5	1	0	11	11	2	1	3	0	287
Unknown	0	0	0	0	0	0	0	0	0	0	0	2

*ARV: antiretroviral (drug); NNRTI: non-nucleoside/non-nucleotide reverse transcriptase inhibitor; NRTI: nucleoside/nucleotide reverse transcriptase inhibitor; PI: protease inhibitor; NVP: nevirapine; EFV: efavirenz.

Table S2: Antiretroviral drugs detected by drug class among samples that were classified as MAA negative using the multi-assay algorithm solely on the basis of HIV viral load ≤ 400 copies/ml*.

	Any ARV	Any NNRTI	Any NRTI	Any PI	NVP only	EFV only	NNRTI only	NRTI only	PI only	NNRTI + NRTI only	All three classes	No. samples
Total Number	57	51	50	4	2	4	6	5	1	42	3	88
Gender												
Female	44	40	38	4	2	3	5	3	1	32	3	65
Male	12	10	11	0	0	1	1	2	0	9	0	22
Unknown	1	1	1	0	0	0	0	0	0	1	0	1
Study site												
Zimbabwe	7	7	5	0	2	0	2	0	0	5	0	8
Tanzania	2	2	2	0	0	0	0	0	0	2	0	6
Vulindlela	39	35	36	1	0	3	3	4	0	31	1	59
Soweto	9	7	7	3	0	1	1	1	1	4	2	15
Age												
≤ 25 years	22	19	18	2	1	2	3	2	1	15	1	43
> 25 years	35	32	32	2	1	2	3	3	0	27	2	45
Subgroup												
Female ≤ 25	18	16	14	2	1	2	3	1	1	12	1	33
Female > 25	26	24	24	2	1	1	2	2	0	20	2	32
Male Age ≤ 25	4	3	4	0	0	0	0	1	0	3	0	10
Male Age > 25	8	7	7	0	0	1	1	1	0	6	0	12
Unk gender >25	1	1	1	0	0	0	0	0	0	1	0	1
Past pregnancy												
Yes	0	0	0	0	0	0	0	0	0	0	0	4
No	44	40	38	4	2	3	5	3	1	32	3	60
Unknown	0	0	0	0	0	0	0	0	0	0	0	1

* ARV: antiretroviral (drug); NNRTI: non-nucleoside/non-nucleotide reverse transcriptase inhibitor; NRTI: nucleoside/nucleotide reverse transcriptase inhibitor; PI: protease inhibitor; NVP: nevirapine; EFV: efavirenz; Unk: unknown.
